# P-314. A Retrospective Review of Long-Acting Lipoglycopeptide Utilization at a VA Tertiary Teaching Medical Center

**DOI:** 10.1093/ofid/ofae631.517

**Published:** 2025-01-29

**Authors:** Megan B Maree, Bonnie Chan, Tiffany Goolsby, Nora T Oliver

**Affiliations:** Atlanta VA Medical Center, Atlanta, Georgia; Atlanta VA Medical Center, Atlanta, Georgia; Atlanta VAMC, Atlanta, Georgia; Atlanta VA Medical Center, Atlanta, Georgia

## Abstract

**Background:**

Dalbavancin and oritavancin are long-acting lipoglycopeptides (LA-LGPs) approved by the Food and Drug Administration (FDA) for acute skin and soft tissue infections (ASSTIs). With their long-acting pharmacokinetics and broad activity against gram-positive bacteria, LA-LGPs are ideal options for patients with serious infections, such as bone, joint, and bloodstream, who (1) require prolonged antimicrobial therapy, (2) are not candidates for long-term intravascular catheter access, (3) prefer to avoid hospital admission, and/or (4) need early discharge.

**Methods:**

A retrospective chart review was conducted on patients ages older than 18 with a medication order of dalbavancin or oritavancin from September 2020- August 2023 at the Atlanta VA Medical Center. Patients were excluded if the patient did not have an active infection or a LA-LGP was ordered but not administered. Pertinent demographic, clinical, and microbiological data were collected from the patient’s medical record.

**Results:**

Resolution of infection in 3 months with LA-LGPs was found to be 76% (35/46) effective in lower extremity osteomyelitis and 74% (72/98) effective for all indications. The mean total length of stay (inpatient) after LA-LGP infusion was 3.9 days. The most common adverse events were nausea and vomiting, which occurred at a rate of 6.1% (6/98).

**Conclusion:**

The utilization of LA-LGPs showed to be effective in infection resolution in a 3-month period in majority of patients. Specifically, lower extremity osteomyelitis, a non-FDA approved use, showed promising results in resolution of infection. The use of LA-LGPs can be beneficial in shortening hospital length of stay and prevention of hospitalizations with minimal adverse events. This can possibly lead to decreased health care cost and hospital acquired infections. In all, the use of LA-LGPs has significant potential use in other serious infections other than ASSTIs with a variety of benefits from the patient and healthcare perspective.

**Disclosures:**

**All Authors**: No reported disclosures

